# “There is no other option; we have to feed our families…who else would do it?”: The Financial Lives of Women Engaging in Sex Work in Ulaanbaatar, Mongolia

**DOI:** 10.5539/gjhs.v5n5p41

**Published:** 2013-05-24

**Authors:** Laura Cordisco Tsai, Susan S. Witte, Toivgoo Aira, Marion Riedel, Hyesung Grace Hwang, Fred Ssewamala

**Affiliations:** 1 Global Health Research Center of Central Asia and Social Intervention Group, Columbia University, New York, USA; 2 Wellspring NGO, Ulaanbaatar, Mongolia; 3 Columbia University School of Social Work, New York, USA

**Keywords:** sex work, women, financial management, HIV/AIDS

## Abstract

**Introduction:**

This article provides an overview of the financial lives of women (n = 204) engaging in sex work in Ulaanbaatar, Mongolia.

**Methods:**

This paper presents findings from a computer-based, interviewer-administered baseline assessment administered with women recruited for participation in a randomized controlled trial testing the feasibility of a combined HIV risk reduction and savings-led microfinance intervention for women engaging in sex work in Mongolia.

**Findings:**

Findings demonstrate that most women are the primary financial providers for their households, using an array of earning strategies to provide for themselves and other dependents, with sex work often constituting the primary household income source. Financial instability in the lives of people engaging in sex work may increase their risk for HIV and STIs due to a compromised ability to negotiate safer sex with partners in times of economic crisis or need. High levels of financial responsibility for household welfare, when combined with low reported savings, the presence of debt, higher premiums offered for sex without a condom, and high levels of harmful alcohol use, may heighten women's risk for HIV and other STIs.

**Conclusion:**

Further research that documents the financial lives of people working in sex work is needed in order to understand the complex relationship between financial stability and engagement in sex work, and to inform the development and testing of structural HIV prevention interventions which target the economic determinants of risk. These findings highlight the importance of economic support programming for women engaged in sex work in Mongolia at a time of rapid economic change in Mongolia.

## 1. Introduction

Given the connection between poverty, gender inequality and HIV risk, structural interventions addressing the social and environmental determinants of risk are critical for HIV prevention efforts (Dworkin & Blankenship, 2009; Gupta, Parkhurst, Ogden, Aggleton, & Mahal, 2008; Kim, Pronyk, Barnett, & Watts, 2008; Sherman, German, Cheng, Marks, & Bailey-Kloche, 2006). While the environmental factors impacting HIV risk are widely acknowledged, structural approaches to HIV prevention have been understudied (Sumartojo, Doll, Holtgrave, Gayle, & Merson, 2000). Scholars have highlighted the need for broader HIV prevention interventions which target the economic structures which influence risk, characterizing structural prevention interventions as the next wave of HIV research (Dworkin & Blaneknship, 2009; Parker, Easton, & Klein, 2000; Sumartojo, 2000). Integrated economic and HIV prevention programs may have great potential for sexual risk reduction among women engaged in sex work (Dworkin & Blaneknship, 2009; Gupta et al., 2008; Kim et al., 2008; Sherman et al., 2006; Kim & Watts, 2005; Parker et al., 2000).

Economic vulnerability is not only a push factor which may impel people to enter sex work, but may also impact the risk behavior of people who engage in sex work as a means of economic support (Odek et al., 2009; Kim et al., 2008; Busza, 2005; Manopaiboon et al., 2003; Tan Minh et al., 2004; Wojcicki & Malala, 2001). Individuals exchanging sex for money or goods may have limited power to negotiate safe sex practices with paying partners due to higher premiums gained through unprotected sex, the price of condoms, and/or the urgent need for income in times of crisis or immediate need (Odek et al., 2009; Stratford, Mizuno, Williams, Courtenay-Quirk, & O’Leary, 2008; Sherman et al., 2006). Women engaging in sex work who report having debt or other economic hardships have been more likely to report unsafe sex practices and have STI symptoms (Reed, Gupta, Biradavolu, Devireddy, & Blankenship, 2010; Ngo et al., 2007). Debt has also been linked to higher levels of physical and sexual violence among people engaging in sex work (George, Sabarwal, & Martin, 2011; Reed et al., 2010).

It is important to note, however, that the relationship between sex work and economic vulnerability is not a simple one. There can be wide discrepancies in levels of income generated through sex work depending upon the type of sex work (street-based versus venue-based) and type of establishment in which one works (Ngo et al., 2007). Sex work can provide a platform for people to financially support themselves and their families, at times preventing their families from falling into poverty (van Blerk, 2008; Ngo et al., 2007; Busza, 2005). For researchers and practitioners interested in developing economic support and empowerment programs for people engaging in sex work, an understanding of the economic lives, strengths, and challenges of this population is critical in ensuring that such economic programs target the priorities, needs, and desires of this vulnerable population. Research regarding the financial lives of people engaging in sex work is, however, limited in scope.

To examine these issues, this paper will provide an overview of the economic lives of women engaging in street-based sex work in Ulaanbaatar, Mongolia. We will use baseline data from a randomized controlled trial testing the effectiveness of the Undarga project, a combined HIV prevention and savings-led microfinance intervention, in reducing sexual risk behaviors and improving the financial health of women engaging in sex work in Ulaanbaatar (Tsai, Witte, Aira, Altantsetseg, & Riedel, 2011). This study has been implemented in a time of rapid economic change in Mongolia due to the development of two substantial mining projects which present unique opportunities for economic growth, as well as challenges for potentially rising economic inequality within the country (Isakova, Plekhanov, & Zettelmeyer, 2012).

## 2. Methods

### 2.1 Study Design

The parent study from which this baseline data is taken aims to test the feasibility and preliminary efficacy of a combined 4-session HIV sexual risk reduction (HIVSRR) and microfinance intervention (including 34 training session and matched savings) to reduce unprotected sex and to decrease proportion of income from sex work among 107 women engaged in high risk sexual activity in Ulaanbaatar, Mongolia. Eligibility criteria for the study included the following: 1) at least 18 years of age; 2) women reported having engaged in vaginal or anal sexual intercourse in the past 90 days in exchange for money, alcohol or other goods; 3) women reported having engaged in unprotected vaginal or anal sexual intercourse in the past 90 days with a paying sexual partner; and 4) women reported interest in learning about and developing her own small business.

Following screening, eligible women completed the informed consent procedures and a baseline assessment. The Columbia University Morningside and National University of Mongolia Institutional Review Boards approved the informed consent protocol for the study. All women who completed the baseline assessment (n = 204) were invited to participate in study sessions. A total of 107 women attended the first study session and were randomized to one of 2 study conditions: 1) the combination HIV sexual risk reduction plus microfinance (HIVSRR+MF); or 2) a 4-session HIVSRR alone control condition.

### 2.2 Sampling

The study employed a targeted sampling strategy in Ulaanbaatar and the peri-urban ger district.^1^ Targeted sampling is a purposive, but systematic method for sampling hard to reach or hidden populations, which is widely used in intervention research with street-based populations. Our approach to targeted sampling occurred in several steps in advance of and during the study recruitment, as suggested by Peterson et al. (2008) and as outlined below. First, a list of street-based venues or sites at which most women working in sex work in Ulaanbaatar solicit their clients was developed from participants in a former Women's Wellness study (Witte et al., 2011), the Undarga program pilot project (Tsai et al., 2011), and Community Advisory Board (CAB) members. After compiling a list of sites, observations were conducted by the Ulaanbaatar-based study team with assistance from outreach workers. During these site visits, team members observed and recorded the number of women working in sex work at the selected site locations, identifying the five with most frequent activity for the study. The study team used the total number of women observed per site to determine a proportional number of women that should be recruited per site to achieve the total sample. The team adjusted the proportions to oversample from the four small sites in order to reach a broader base of women engaged in sex work. We also inserted a question in the study screening to ask participants about additional sites in Ulaanbaatar where women engage in sex work. The final list of sites from which women were recruited totaled 10 sites, including 5 additional sites identified by women participating in the study.

After all site observations were completed, Wellspring NGO staff and peer outreach workers recruited women at the study sites mentioned above. Recruiters spent several minutes describing the project to the potential participants. For those who expressed interest in participation, recruiters then screened participants for their eligibility. Only women who expressed interest in participating in the study and met study eligibility criteria were invited to participate in the baseline assessment.

### 2.3 Data Collection

All participants completed a computer-based baseline assessments administered by trained interviewers. Interview questionnaires were developed in collaboration between the Mongolia based and U.S. based research teams. All items had been pre-tested with women engaged in sex work in Ulaanbaatar prior to being administered to study participants. Assessments were conducted in a private room at the Wellspring NGO office and in a privately rented room at one of the sex work sites identified through the targeted sampling process. Women received 6000 MNT^2^ for participating in the baseline interview. After completing the baseline assessment, women were invited to attend the first intervention session. This paper presents descriptive results from the study baseline data only describing financial variables and the economic context of participants’ lives.

### 2.4 Measures

#### 2.4.1 Household Earnings

At baseline, several questions were asked to understand household earnings. We asked participants “on average, what is the total monthly income for your entire household (including income from all household members and all sources)” and “on average, how much is your total monthly income (money that you earn, not from other members of your household).” Women were asked “on average, how much of your monthly income is from sex work?” As a secondary measure, we also asked “over the past month, what percentage of your income has come from sex work?” To understand more about the women's engagement in sex work, the study team asked “is exchanging sex for money your main source of income” (yes/no). We requested that women note the top three ways they earn money (which may or may not include sex work), as well as the months of the year in which they earn money (from sex work and activities apart from sex work). Women were also asked “how often would you say you are offered more money in exchange for sex without a condom?”

Additionally, the study team asked questions about the number of income earners and financial dependents in the women's households. Women were asked to report the other income earners in the household and the total number of people in the household who earn money. The study team asked the women how many children they have given birth to who currently depend upon them for financial support on a monthly basis, and how many additional people depend upon them for the majority of their food and shelter. We also created a new variable – estimated income per person – which was created by dividing the average woman's monthly income by the number of her financial dependents (herself plus all minor and adult dependents). We used this estimate to determine the number and percentage of women living below the poverty line.

#### 2.4.2 Savings

We asked women: “Do you currently have money saved anywhere?” If they reported yes, women were asked how much they currently have in savings and where they currently save money. We created a new variable – savings as a percentage of women's average monthly income – by dividing the amount in current savings by women's average monthly income. All women were asked: “Do you have a specific savings goal?” If yes, we asked women to share their savings goal(s); options included education for self, education for children, business development, housing, health care, emergencies, general household expenses, and other. We also asked: “if you had 5,000 MNT, what would you do with it?” Options included “spend all of it, spend most of it, spend some and save some, save most of it or save all of it.”

#### 2.4.3 Debt

Women were asked “do you currently owe anyone money?” If yes, women were asked: “in total, how much money do you currently owe,” “who do you owe money to,” “what are the top two expenses that you borrow money for?” and “how much money do you usually owe?” We created a new variable – percentage of income usually owed – by dividing the amount women usually owe by women's average monthly income.

#### 2.4.4 Intimate Partner Abuse and Economic Security

The study team incorporated two questions about financial self-efficacy and financial security and future safety from the Domestic Violence-Related Financial Issues Scale (DV-FI) (Weaver, Sanders, Campbell, & Schnabel, 2009). First, we asked: “how confident are you that you can meet your goals for becoming financially secure?” Secondly, we asked the women to rate the following on scale from strongly disagree to strongly agree: “adequate employment would be helpful in increasing my current and future safety.” A series of questions on economic abuse and the perceived financial role in partner abuse from the DV-FI was also included (Weaver at al., 2009). We asked women to rate the following from very strongly disagree to very strongly agree: “financial insecurity has played a role in my previous experience of partner violence;” “inability to save has played a role in my previous experiences of partner violence;” “lack of adequate employment has played a role in my previous experiences of partner violence;” and “debt has played a role in my previous experiences of partner violence.” Women were asked to answer yes or no to indicate whether they agree with the subsequent statements: “my trust partner has prevented me from having access to money;” “my trust partner negatively affected my debt;” “my trust partner prevented me from obtaining education;” and “concerns or worries about my financial future affect my decisions about whether to stay with my trust partner or leave my trust partner.”

#### 2.4.5 Focus Group Discussions

Two focus group discussions (FGDs) were held with 8 participants who reported having trust partners to clarify the nature of the women's economic relationships with their trust partners.

#### 2.4.6 Alcohol Use

Additionally, the study team calculated Alcohol Use Disorders Identification Test (AUDIT) scores for all participating women. The AUDIT is a ten-item instrument used to identify harmful alcohol consumption (Babor, Higgins-Biddle, Saunders, & Monteiro, 2001).

### 2.5 Data Analysis

The study team calculated simple frequencies and percentages for all measures described above from the baseline interviews. The study team did not conduct a systematic qualitative analysis of the FGD transcripts; quotations were included in the paper for illustrative purposes only.

## 3. Results

### 3.1 Socio-Demographic Characteristics

Participating women ranged from age 18 to 61, with a standard deviation of 9.37 years. The mean age of women in the sample was 35.55 years, with a mode of 32 years. The vast majority of women (92.16%) of the women have completed secondary schooling or beyond, with 8.82% of women having graduated from a 4-year college or university. A total of 51.47% currently have a trust partner. One hundred and seven women (52.45%) are divorced, 10.78% are currently married, and the remainder has never been married. Most (85.78%) of the women have children. Over half of the women have either 1 or 2 children (27.94% and 23.04% respectively); almost one fifth of the women (18.14%) have four or more children.

The vast majority of women (95.59%) report engaging in sex work without a pimp or a “boss.” The mean number of years women have been engaged in sex work is 6.18 years, with a mode of 3 years and a standard deviation of 5.38 years. Approximately half of the women (50.49%) have been working in sex work for 4 years or less. Twenty-two women (10.78%) have been working in sex work for fifteen years or longer. The average age of entry into sex work was 29.17 years, with the age of entry ranging from 14 to 52 and a standard deviation of 8.50 years.

### 3.2 Household Earnings

As reflected below in Table 1, most women reported sex work as their primary source of income. Eighty-one women (39.71%) personally earn on average less than 250 000 MNT monthly (184 USD), and 9.80% earn more than 1 000 000 MNT per month (737 USD). On average, 21.57% have a total household income of less than 250 000 MNT monthly, 42.65% have a household income from 250 000 – 500 000 MNT monthly, 23.53% have a household income from 500 000 – 1 000 000 MNT monthly, and 12.25% have a household income of more than 1 000 000 MNT per month. Approximately one quarter of the women have no children who depend on them financially (25.98%). Half of the women have 1-2 dependent children (54.90%), and remainder has 3 or more (19.12%). The women also, on average, have 1 other adult who is financially dependent upon them. According to the Mongolia National Statistics Office (2012), the poverty line in Ulaanbaatar is 126 500 MNT per person. Table 1 reflects the estimated percentage of women and their households below the poverty line.

**Table 1:** Participant reports of household earnings, financial dependents, income from sex work, and enhanced risk due to sex work

Variable	Total sample (n = 204)
	Percentage (n)
Sex work as woman's main source of income	
Yes	87.75% (179)
No	12.25% (25)
Percentage of woman's income from sex work
< 50%	4.90% (10)
50 – 74%	11.76% (24)
75 – 100%	83.33% (170)
Estimated income per head
< 126 500 MNT (below poverty line around 93 USD)	60.78% (124)
≥ 126 500 MNT (at poverty line or above around 93 USD)	39.22% (80)
Primary income generator in household
Respondent herself	78.93% (161)
Her trust partner	4.90% (10)
Another adult relative	9.31% (19)
One of the respondent's children	2.94% (6)
Other	3.92% (8)
Frequency of being offered more money for sex without a condom
Never	3.92% (8)
Less than half the time	42.16% (86)
Half the time	20.10% (41)
More than half the time	33.82% (69)

One hundred and thirteen women (55.39%) stated that they earn money from sex work every month of the year. The other women engage in sex work only part of the year, with involvement in sex work highest during the warmest months of the year (May – August). One hundred and seventeen women (57.35%) report that their only source of income is sex work; the rest of women report engaging in a number of different means of earning income, mixing sex work with other income sources. The most common ways of obtaining money outside of sex work include receiving government grants (19.61% of the total sample), working in a restaurant (6.37%), running a small business (4.41%), construction work (3.43%), cleaning (2.45%), and childcare (1.47%).^3^ These government grants include revenues from mineral deposits distributed to all Mongolian citizens, as well as need-based social welfare grants to families below the poverty line. Women reported that over the past three months, they have exchanged sex for money or goods with an average of 91 people.

### 3.3 Savings

As reflected in Table 2, most women reported having no savings. For the women who reported having savings, 6.37% of the total sample holds less than 500 000 MNT in savings (approximately 368 USD), 6.86% have 500 000 – 1 000 000 MNT (368 – 737 USD) and 2.94% have over 1 000 000 MNT (737 USD) in savings. When asked what they would do with 5000 MNT, 68.14% of women reported they would spend all of it, 8.33% reported they would spend most of it, 16.18% reported they would save some and spend some, 2.94% reported they would save most of it, and 4.41% reported they would save all of it.

A total of 64.22% of the entire sample reported having savings goals, even though only 16.18% of the entire sample reported that they currently had money in savings. The remaining 35.78% reported having no savings goals. Women could report multiple goals for their savings. Among those who reported having savings goals, the women's savings goals were as follows: 54.20% reported their children's education, 28.24% reported general household expenses, 11.45% reported housing, 9.92% reported health care, 6.11% reported emergencies, 5.34% reported business development, and 3.82% reported their own education. Other goals included general saving for their future and their children's futures, debt repayment, and their siblings’ education.

**Table 2:** Participant report of savings, savings as a proportion of income and location of savings

Variable	Total sample (n = 204)
	Percentage (n)
Whether women currently have any savings
Yes	16.18% (33)
No	83.82% (171)
Current savings as percentage of women's average monthly income
0%	83.82% (171)
< 50%	3.92% (8)
50 – 100%	2.45% (5)
> 100%	9.80% (20)
Where women save
No savings	83.82% (171)
Bank account	13.24% (27)
At home	1.47% (3)
Other	1.47% (3)

### 3.4 Debt

As reflected below in Table 3, just over half of the women currently owe money. Women reported borrowing money for numerous expenses simultaneously. Among those who borrowed, 63.30% of women reported borrowing money for day-to-day living expenses, 13.76% reported borrowing money for health care, 11.93% reported borrowing money for education, 5.50% reported borrowing for non-health related emergencies, 4.59% reported borrowing money for other peoples’ debts, and 3.67% reported borrowing money for employment-related expenses. Women also reported borrowing money for alcohol and larger purchases such as mobile phones and computers. Women were also asked all the places and people from whom they borrow money. Among the women who have debt, 29.36% take store credit, 30.28% reported borrowing from friends and/or neighbors, 13.76% borrow money from a moneylender, 11.93% borrow from formal lending institutions, 9.17% borrow from family members, 7.34% borrow from their landlord, and 1.83% borrow from their ‘boss’ or pimp. Nine women (8.26%) report borrowing from other sources, including electricity service providers and hotels.

**Table 3:** Participant reports of debt, amount of debt and debt as a percentage of income

Variable	Total sample (n = 204)
	Percentage (n)
Does the woman currently owe anyone any money?
Yes	53.43% (109)
No	46.57% (95)
Amount women currently owe
0	46.57% (95)
< 100 000 MNT (< 74 USD)	18.63% (38)
100 000 – 499 999 MNT (74 – 367 USD)	21.08% (43)
500 000 – 999 999 MNT (368 – 736 USD)	5.39% (11)
≥ 1 000 000 MNT (≥ 737 USD)	8.33% (17)
Amount women usually owe
0	28.43% (58)
< 100 000 MNT (< 74 USD)	51.96% (106)
100 000 – 499 999 MNT (74 – 367 USD)	16.18% (33)
≥ 500 000 MNT (≥ 368 USD)	3.43% (7)
Percentage of women's income usually owed
0	28.43% (58)
< 25%	50.49% (103)
25 – 49%	9.80% (20)
50 – 100%	6.86% (14)
> 100%	4.41% (9)

### 3.5 Intimate Partner Abuse and Economic Security

In general, women reported high financial self-efficacy scores. Under half of the women (41.67%) reported that they are “confident in general” about their ability to meet their goals for becoming financially secure. The second highest group (30.39%) reported being “truly confident” in their abilities; only 1.47% reported that they are “not at all confident” in their abilities. A total of 91.67% of the women reported that they very strongly agree (41.67%), strongly agree (37.75%) or somewhat agree (12.25%) that adequate employment would increase their current and future safety.

Most women disagree that financial issues have played an important role in their previous experiences of intimate partner violence (IPV). Consistently, approximately one fifth of women believe that economic issues were important in their IPV experiences. Twenty-four women (22.86%) reported that they very strongly or strongly agreed that lack of adequate employment played a role in their previous IPV experiences. Eighteen women (17.14%) reported that they very strongly/strongly agreed that financial insecurity played a role in their previous IPV experiences; 16.19% reported that they very strongly/strongly agreed that an inability to save played a role in their previous IPV experiences; and 18.10% of women reported that very strongly/strongly agreed that debt played a role in their previous IPV experiences. Twenty-eight women (26.67%) reported that their trust partner has prevented them from accessing money, 24.76% reported that their trust partner has negatively impacted their debt, and 14.29% reported their trust partner has prevented them from obtaining education. Forty-eight women (45.71%) reported that worries about their financial futures affect their decisions of whether or not to stay with their trust partners.

### 3.6 Alcohol Use

Almost half of the women (44.61%) scored in the highest risk level (Zone IV) in regard to their alcohol use. Only 44 women (21.57%) scored in the lowest risk level (Zone I).

## 4. Discussion

This study examines the financial lives of women engaged in sex work in Mongolia in order to better inform the development of structural interventions that may achieve both economic empowerment and HIV risk reduction. Many of the women participating in this study have obtained high levels of education and report high financial self-efficacy. At the same time, 63% of women also report that they originally entered sex work due to financial difficulties or family financial crises, with many indicating that they were unable to find other kinds of employment. The lived realities for these women are situated within a broader context of devastating economic changes in Mongolia since 1990 due to Mongolia's transition to a market economy. This economic restructuring has had a disproportionately adverse impact on women in Mongolia, as state sector employment has decreased, state entitlements have diminished, and women have faced increasing challenges obtaining employment in the formal sector while primarily shouldering family caregiving responsibilities (UNIFEM, 2001). Presently, the Mongolian economy is also in a state of rapid change. The Mongolian economy is currently one of the fastest growing economies in the world, in part due to the development of the Oyu Tolgoi and Tavan Tolgoi mines. This growth is, however, contributing to high inflation and rising food prices, adversely impacting those living in poverty (World Bank, 2012). While the development of the mining industry is predicted to generate an economic boom in Mongolia over the upcoming years, there are also concerns that the financial benefits of this boom will only ensue for small proportion of the population, deepening economic inequality in a country which has traditionally had moderate levels of inequality (Isakova, Plekhanov, & Zettelmeyer, 2012). Coupled with recent political instability, this situation leads to a climate of uncertainty for those living near the poverty line, including some women engaging in sex work in Ulaanbaatar.

The women in this study demonstrate great resilience while balancing numerous financial challenges. Few women report having savings and more than half of the women reports having debt – primarily store credit and debt from moneylenders. Most women are the primary income earners in their households with a mode of three people depending upon them for financial support. Anecdotal data from focus groups suggests that women have strenuous economic relationships with their trust partners, with trust partners seemingly adding financial burdens for the women. Participants expressed that their trust partners face their own difficulties obtaining employment, use alcohol to an extent that interferes with their ability to earn, and/or use their limited financial resources to support others outside the women's households (such as extended family networks, multiple trust partners, and/or their own children). Many women expressed a sense of resignation about their situation. As one woman summarized: “There is no other option. We have to feed our families…who else would do it?”

HIV risk among the women in this study is further exacerbated by the fact that sex work constitutes a majority of their income, and that over half of the women are offered more money for sex without a condom half or more than half of the time. These factors suggest that the women's power to negotiate condom use with paying partners may be inhibited in times of family financial crisis or immediate need. Further, most women report very high levels of harmful alcohol use, indicating alcohol abuse and/or dependence issues that in and of themselves pose health risks, but which also further exacerbate and heighten risk for sexual transmission of HIV infection and other sexually transmitted infections (STIs). Women talk about using alcohol to cope with the lifestyle and heavy stigma associated with street sex work. Over time and left untreated, there is the concern that sustained or increased alcohol use will also more severely compromise what economic security they may have from sex work.

## 5. Limitations

Study limitations deserve consideration. First, the limited sample size of the study prevents generalization to a broader population of women engaging in sex work in Mongolia. Data was collected through self-report, and some questions that involved numerical calculations proved challenging for some participants. Several of the measures, such as estimated income per head and percentage of women's income usually owed, were transformed in order to present them in a more accessible manner and therefore must be taken as estimates. These transformed variables assumed equal distribution of resources among household members for the sake of generating a rough estimate, an assumption that is neither realistic nor consistent with existing literature (Lise & Seitz, 2007; Duflo, 2003). Further research is needed to understand the processes of intra-household resource allocation within the households of women working in sex work in Mongolia. Additionally, questions on the women's experiences of IPV within trust partner relationships were not asked. As a result, we were unable to determine whether the women who noted a limited financial role in their previous history of IPV did so because economic factors did not impact their IPV experiences or because they did not experience IPV. Limitations notwithstanding, this study is an important first step in understanding the financial lives of women engaging in sex work in Mongolia and contributes to the growing literature informing structural interventions to address HIV risk.

## 6. Conclusions

In light of the synchronicity of a swelling economy, coupled with persistent high unemployment and risk for increased HIV infection, we believe that Mongolia is well poised to test the feasibility of structural interventions targeting both economic empowerment and HIV prevention. Our findings illuminate the complex financial lives of women exchanging sex, who rely on sex work for income, but also use financial instruments and articulate goals for family survival. Findings underscore the importance of economic support programming for women engaging in sex work in Mongolia to offer HIV risk reduction options that do not require women to choose between safety and family survival. Future economic interventions for this population should consider how to increase the economic stability of women engaging in sex work while considering gendered norms around partnering and financial decision-making. The dual goal of reducing new STIs and increasing economic empowerment may be only one of many other secondary gains to be achieved in such a structural intervention, particularly at this time in Mongolia.

## Footnotes

1 A ger is a circular residence which is constructed with wooden frames and felt walls. Gers have traditionally been used as the dwelling place of nomadic peoples in Mongolia. In urban Ulaanbaatar, residents construct gers in congregated areas called ger districts.

2 At the time of writing this paper, the conversion rate between MNT and USD was 1355 MNT per USD. Due to rapid inflation in Mongolia, the conversion rate changed throughout the project.

3 Several responses were recoded from the “other” category to existing categories, in consultation with the Ulaanbaatar-based research team.
